# Medical Aid in Dying: A Narrative Review of the Recent Academic Literature in the United States

**DOI:** 10.7759/cureus.104134

**Published:** 2026-02-23

**Authors:** Holland Kaplan, Soraira Pacheco, Keziah M Thomas, Christopher L Ulmschneider, Anjiya Sulaiman, Chandni Lotwala, Derek Dawes, Issa A Hanna, Courtney Nguyen, Caroline G Snider, Gabriel M Aisenberg

**Affiliations:** 1 Palliative Care, Baylor College of Medicine, Houston, USA; 2 Geriatrics, University of Texas Health Science Center at Houston, Houston, USA; 3 Internal Medicine, University of Texas Health Science Center at Houston, Houston, USA; 4 Dentistry, University of Texas Health Science Center at Houston, Houston, USA; 5 Palliative Care, Rice University, Houston, USA

**Keywords:** active and passive euthanasia, end-of-life ethics, maid, medical-aid-in-dying, medical-assistance-in-dying

## Abstract

Medical aid in dying (MAiD) is a practice in which a healthcare professional provides assistance to a terminally ill patient seeking to end their life. To assess how academic discourse may shape public opinion and policy in the United States, we conducted a narrative review of the literature published between 2020 and 2024. Articles were categorized as supportive, opposing, or neutral, and patterns were examined across authorship, disciplinary focus, and target populations. Our findings reveal that most recent publications adopt supportive or neutral stances toward MAiD, with a slight increase in opposition beginning in 2022. Authorship discipline strongly influenced position, with legal journals disproportionately supportive and religious journals more frequently opposed. Arguments favoring MAiD emphasized autonomy and relief of suffering, whereas opposing articles highlighted risks to vulnerable populations and potential harm. Academic literature contributes significantly to shaping the national conversation around MAiD and may influence evolving societal attitudes and policy development regarding end-of-life options.

## Introduction and background

Background

Medical aid in dying (MAiD) refers to a medical practice where a healthcare professional provides assistance to a terminally ill patient who wishes to end their life. The American Medical Association (AMA) adopts a resolutely negative stance on this practice, asserting that MAiD would be “fundamentally incompatible with the physician’s role as healer, difficult or impossible to control, and pose serious societal risks [1].” Despite this opposition, MAiD, often framed as a means to uphold patient autonomy, has been legalized in 12 United States (US) jurisdictions: Oregon (1994), Montana (2009), Washington (2009), Vermont (2013), California (2016), Colorado (2016), the District of Columbia (2017), Hawaii (2019), Maine (2019), New Jersey (2019), New Mexico (2021), and Delaware (2025). To qualify for MAiD in the US, a patient must be an adult with decision-making capacity who has been diagnosed with a terminal illness that will result in death within six months. States have various safeguards in place, including requirements that at least two healthcare providers confirm a patient’s eligibility for MAiD, a mental health evaluation be conducted, and that a mandatory waiting period occurs between the request and provision of the medication [2].

Advocacy groups in the US promote MAiD under the ethos that “a society that affirms life and accepts the inevitability of death embraces expanded options for compassionate dying and empowers everyone to choose end-of-life care that reflects their values, priorities, and beliefs [3].” This advocacy takes place against the backdrop of global developments that may influence practice in the US, including the recent legalization of MAiD across Australia (2024-2025), ongoing legislative efforts in the British Parliament, and recent changes in MAiD legislation in Canada, in which an individual’s sole underlying medical condition may be a mental illness [4-6].

Objectives

To better understand the trajectory of academic perspectives on MAiD in the US that may be impacting public opinion and policy development, we conducted a broad literature review of articles published on this topic between 2020 and 2024. By categorizing article perspectives as supportive, opposing, or neutral and analyzing patterns across authorship, disciplinary focus, and target populations, we aimed to characterize the variables impacting the national discourse on MAiD and explore how the academic literature contributes to this ongoing conversation.

## Review

Search strategy

Despite being a narrative review, this study followed the Preferred Reporting Items for Systematic Reviews and Meta-Analyses (PRISMA) guidelines, with the purpose of being comprehensive [7]. We broadly searched PubMed, CINAHL, EMBASE, and Web of Science for publications between January 2020 and December 2024 that addressed MAiD.

Study selection

We used the following Medical Subject Headings (MeSH) terms: “physician-assisted suicide,” “euthanasia,” “physician-assisted death,” “assisted suicide,” “suicide by physician,” and “medical aid in dying.” Additional articles were identified through review of the references. Inclusion criteria were: 1) English language; 2) at least one author with a US institutional affiliation; and 3) primary focus on MAiD. Articles that did not meet these three characteristics were excluded from the analysis.

Categorization

Retrieved articles were divided among five pairs of investigators, who independently assessed eligibility and categorized each article as supportive, opposed, or neutral toward MAiD. Disagreements were resolved by consensus of the 10-investigator team. Articles were classified as 1) supportive, if the author(s) clearly emphasized the benefits of MAiD over its harms; 2) opposed, if they emphasized harms over benefits; or 3) neutral, if they were solely descriptive or if they emphasized the benefits and harms equally. For survey studies, classification was based on the prevailing view of the survey respondents rather than the authors’ views. Each included article was further characterized by: 1) number of authors (single versus multiple); 2) journal type (medical, religious, legal, or ethical/moral); 3) article type (position, survey, review, observational, workshop, or experimental); 4) population focus (physicians, residents, nurses, general population); and 5) whether the article originated in a state where MAiD is legal. We defined “position” articles as those expressing authors’ opinions and “review” articles as those involving a literature review, even if not systematic. 

Statistical analysis

Predictors of supportive versus opposing positions were analyzed using Fisher’s exact test, with p<0.05 considered statistically significant.

Results

**Figure 1:**
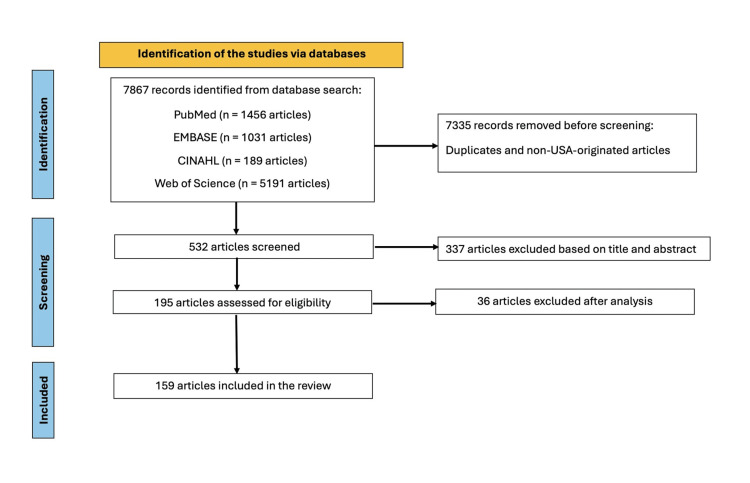
Preferred Reporting Items for Systematic Reviews and Meta-Analysis (PRISMA) flow diagram

Figure 1 shows the PRISMA flow diagram.

A total of 159 articles met the inclusion criteria [8-166]. Among them, 69 (44%) supported MAiD, 23 (14%) opposed MAiD, and 67 (42%) were neutral. Table 1 summarizes article characteristics.

**Table 1:** Summary of article characteristics (n=159) (#) clear for 158 articles; (*) clear for 152 articles; MAiD: medical aid in dying

			References
Position	Supportive	69 (44%)	[11,12,16-18,21,23,24,26,30,32,34,35,40,43,46,48,52,54,55,63,65-70,72-74,76,79,80,82,87, 88,94,101,103,117-119,122-126,129,131,133,134,135,137,139,140,142,143,148-150,153-155,158,160,161,164-166]
	Opposing	23 (14%)	[9,10,19,20,22,41,42,49,50,57,60,71,75,81,83,84,104,106,108,112,114,128,156]
	Neutral	67 (42%)	[8,13-15,25,27-29,31,33,36-39,44,45,47, 51,53,56,58,59,61,62,64,77,78,85,86,89-93,95-100,102,105,107,109-111,113,115,116,120,121,127,130,132,136,138,141,144-147,151,152,157,159,162,163]
Article type	Position	75 (47%)	[10,12,16-20,22,23,30,32,35,36,40,42,43,46,49,50,54,57,60,64,66-83,85-89,93,94,96, 98,104,106-108,112,114,116-119,123,128,136,137,139,140,145,148-150,152,153,155,156,158-160]
	Survey	40 (25%)	[8,11,13-15,21,26,28,29,33,34,37-39,41,45,51-53,58,59,61,65,77,95,99,100,102,103,105,109,110,122,130,132,135,142,147,157,163]
	Review	21 (13%)	[9,27,31,47,84,90-92,97,101,121,126,131,141,151,154,161,162,164-166]
	Observational	20 (13%)	[24,25,44,48,55,56,62,63,111,113,115,118,120,124,125,127,129,133,134,146]
	Workshop	2 (1%)	[143,144]
	Experimental	1 (1%)	[138]
Journal type #	Medical	82 (52%)	[8,11,13,15-18,20,24-31,34,37,38,44,45,48-53,56,58,59,61,62,65,77,89,90,94-103,105,109-111,114,117,118,120-122,124,125,127,129,130,132-140,142-149,151,160,164,165]
	Ethics	49 (31%)	[10,12,23,33,36,39-41,43,46,47,54,57,63,67-73,75,78-88,91,93,112,113,119,123,131,152,154-157,159,161-163]
	Religious	13 (8%)	[9,14,19,22,35,42,60,104,106,108,126,128,153]
	Legal	9 (6%)	[21,32,55,74,76,107,115,150,166]
	Other	4 (3%)	[64,66,92,116]
Focus *	Multiple	83 (55%)	[8-14,16-33,35,36,39-43,51,54,55,60,62,64,78-92,95, 97-99,101,103-110,112-116,119,123,126,128-132,136-138,163]
	Population	45 (30%)	[44,46-48,56,57,59,66-77,127,140-156,158-162,164-166]
	Attending	16 (11%)	[15,34,37,38,45,49,50,52,53,61,63,100,118,120,121,157]
	Nurses	6 (4%)	[102,117,124,125,133,134]
	Residents	2 (1%)	[58,65]
Number of authors	One	64 (40%)	[9-12,14,19,20,23,27,29-31,35,39,42,43,46,47,50,54,60,68-72,76,79,82-84,86,87,91,94,101,104-106,108,112,113,116,117,119,126,128,129,131-133,136,137,149,152,153,155,158-161,164-166]
	More than one	95 (60%)	[8,13,15-18,21,22,24-26,28,32-34,36-38,40,41,44,45,48,49,51-53,55-59,61-67,73-75,77,78,80,81, 85,88-90,92,93,95-100,102,103,107,109-111,114,115,118,120-125,127,130,134,135,138-148,150,151,154,156,157,162,163]
	From multiple states	46 (29%)	[8,13,15,17,18,21,22,25,34,36,40,44,45,49,51,52,56,65,66,73,74,77,78,88-90,97-99,109,114,115,118,123-125,127,130,138,141,145,148,154,156,157,163]
	At least one state where MAiD is legal	29 (18%)	[13,15,17,18,21,25,34,44,45,51,52,56,73,78,88-90,98,123-125,127,130,138,145,148,154,157,163]

Sixty-four (40%) were single-authored. Of the 95 (60%) articles with multiple authors, 46 (29%) included contributors from different states, and 29 (18%) had at least one author based in a state where MAiD was legal. Overall, 57 (36%) articles originated in states with MAiD legislation.

**Figure 2:**
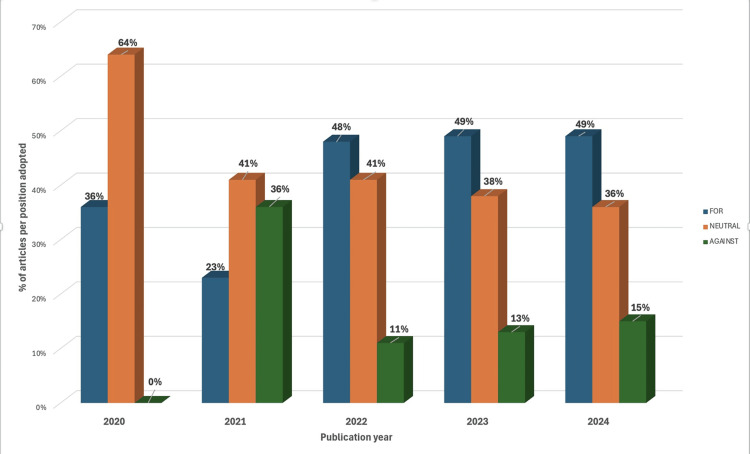
Years of publication of the selected articles and the articles’ positions regarding medical aid in dying

From 2020 to 2024, the distribution of article positions shifted. In 2020, 14 (64%) publications were neutral, and eight (36%) expressed support, with no opposition. By 2022, articles with supportive positions had increased to 14 (48%), while neutral stances declined to 12 (41%). In the subsequent years (2023-2024), the distribution remained stable with a slight increase in opposing articles to six (15%) by 2024 (Figure 2).

Supportive articles, compared to those that were neutral or opposing, were more likely to appear in legal journals (7 (10%) vs. 2 (2%), P=0.04), to be position papers (40 (58%) vs. 35 (39%), P=0.02), and focus on the general population rather than healthcare professionals (26 (38%) vs. 19 (21%), P=0.03). Conversely, publication in religious journals was strongly associated with opposition to MAiD (9 (39%) vs. 4 (3%), P<0.001). Surveys were more often neutral or opposed to MAiD than supportive of the practice (30 (33%) vs. 10 (14%), P=0.009). However, surveys including physicians were more likely to adopt a supportive position, with an overall positive opinion among 1391 (66%) physicians, versus 705 (34%) negative opinions (total interviewed: 2096 physicians) [8,15,26,34,52,109,130]. 

Table 2 provides examples of phrases characterizing supportive, neutral, and opposing positions regarding MAiD [11,31,43,97,108,156].

**Table 2:** Examples of phrases suggestive of a supportive, neutral, and opposing position to medical aid in dying MAiD: Medical aid in dying; PAD: physician aid in dying

	Examples of phrases
Supportive	“Our study complicates simple notions of aid-in-dying as a person’s choice and way to maintain one’s dignity. Anchored values like control and choice competed with other valued aspects of their lives, including their loved ones, spirituality, and the meaning attached to living another day [11].”
	“If granting a request for PAD in the case of bodily illness (like metastatic cancer) is sometimes permissible, then, I argue, granting requests for PAD in the case of severe, treatment-resistant depression is also sometimes permissible [43].”
Neutral	“Enduring controversies about the general ethical permissibility of MAiD, the relationship of MAiD to palliative care and hospice, when physicians should promote alternative interventions, and the effect of psychiatric and cognitive symptoms on access to MAiD continue to inform societal discourse and legal responses to the practice [31].”
	"According to Gallup, Americans’ support for painless death for the incurable shifted from 37% in 1947 to 75% in the early 1990s [97]."
Opposing	“Christians have an obligation to advocate for people with disability who view PAS as against their dignity [108].”
	“The phenomenon of the slippery slope is, in large part, the expectable consequence of 'normalizing' or naturalizing the physician’s direct or indirect killing of the patient via euthanasia or PAS, respectively. The more widely these acts are performed, the easier it becomes to mischaracterize them as forms of “medical care” [156].

Discussion

Since 2022, our analysis shows a balanced distribution of supportive and neutral positions towards MAiD in academic literature. Opposing views have slowly increased. This contrasts with the AMA’s categorical opposition and points to a more nuanced academic discussion than in official policy statements. The slow rise in opposing views since 2022 may reflect international and political developments. These include controversies over mental health as a qualifying condition in Canada and increased legislative activity in the US, which have led to more polarized views [166]. In 2024 and 2025, four US states (New York, Massachusetts, Illinois, and Virginia) failed in efforts to legalize MAiD [167-170]. International events, such as the first use of the “Sarco suicide pod” in Switzerland, have also reignited the MAiD debate [171].

In our analysis, single-author papers more often demonstrated supportive positions. Individual authors may feel more empowered to express opinions about MAiD than groups of authors or larger professional organizations. The latter may need to reach a consensus or reflect institutional priorities before releasing statements. In terms of disciplinary focus, articles published in legal journals were more often supportive of MAiD and focused on autonomy. Conversely, articles in religious journals were commonly opposed to MAiD, with arguments often rooted in the concept of the sanctity of life. Articles targeting the general population tended to be more supportive of MAiD, possibly reflecting a societal preference for autonomy in end-of-life decisions. Notably, this preference is not fully mirrored by professional medical organizations [172]. This mismatched trend between patient preferences and medical organizations has been reported in European countries for over a decade [2,173].

Survey-based articles captured the views of healthcare professionals, members of professional medical societies, or the general public. Although surveys overall included more respondents opposing MAiD, those that surveyed physicians revealed predominantly supportive views. These studies are critical given that most US medical and surgical societies have not issued formal positions on MAiD. A recent review found that only 11 such societies had done so, with 5 opposing and 4 supporting [62].

Our review revealed several common arguments both in favor of and against MAiD. Supportive arguments emphasized three themes: respect for patient autonomy in end-of-life decisions, a duty to alleviate suffering when curative treatments are exhausted, and evidence of MAiD’s safety and efficacy where it has been implemented. Patients and caregivers who participate in MAiD often express appreciation and satisfaction with the support and services provided. Critics of MAiD focus on risks to vulnerable populations, particularly those with reversible psychiatric conditions or inadequately addressed pain. Those opposing MAiD also point out that effective palliative care can improve comfort without resorting to MAiD [174].

Our study has several limitations. First, while all papers were treated equally in the analysis, publications vary in influence: editorials or opinion pieces represent the view of fewer authors, while surveys represent broader professional and public sentiment. Articles published in high-impact journals and those that are widely cited may carry proportionally more influence in the academic discourse. Additionally, our literature review did not capture large, public surveys of the general population (e.g., those conducted by Gallup or Pew Research Center) [175]. Our study is solely intended to reflect perspectives and data in the academic literature. Second, the studies focused on the population at large are not explicit enough to define what segments of the population they included. For instance, it is not clear if they deliberately excluded healthcare workers or whether the participants had experience with MAiD. Third, surveys include answers at a specific time, whereas the opinions regarding controversial topics tend to fluctuate in time, as they are influenced by contextual circumstances. Perhaps, re-addressing the surveyees' opinion time after MAiD occurred could offer clarification in this regard.

## Conclusions

This narrative review demonstrates trends in the academic perspectives on MAiD in the US between 2020 and 2024. While the official positions of many medical organizations, such as the AMA, remain opposed to MAiD, the academic literature reveals predominantly supportive and neutral perspectives with a mild rise in opposition since 2022. Our findings also suggest that authors’ disciplinary backgrounds influence their published positions, with articles in legal journals more likely to be supportive of MAiD and articles in religious journals more likely to oppose MAiD.

Arguments in favor of MAiD continue to focus on autonomy and relief of suffering, while opposing arguments center on risk of harm, particularly to vulnerable patients. As end-of-life options expand in the US and societal attitudes evolve, the academic literature plays an important role in framing the conversation about MAiD.
